# Trans-vaccenic acid inhibits proliferation and induces apoptosis of human nasopharyngeal carcinoma cells via a mitochondrial-mediated apoptosis pathway

**DOI:** 10.1186/s12944-019-0993-8

**Published:** 2019-02-09

**Authors:** Jian Song, Yujie Wang, Xiaoqin Fan, Hanwei Wu, Jinghong Han, Ming Yang, Lu Lu, Guohui Nie

**Affiliations:** 1 Department of Otolaryngology, Shenzhen Second People’s Hospital, The First Affiliated Hospital of Shenzhen University, Shenzhen, 518035 People’s Republic of China; 2 Institute of Translational Medicine, Shenzhen Second People’s Hospital, The First Affiliated Hospital of Shenzhen University, Shenzhen, 518035 People’s Republic of China; 3 Department of Otolaryngology, Peking University Shenzhen Hospital, Shenzhen, 518036 People’s Republic of China; 4 Department of Otolaryngology, Shenzhen People’s Hospital, The second Affiliated Hospital of Jinan University, Shenzhen, 518000 People’s Republic of China

**Keywords:** Trans-vaccenic acid, Apoptosis, Nasopharyngeal carcinoma, Akt, Bad, Mcl-1

## Abstract

**Background:**

Intake of trans fatty acids (TFAs) from partially hydrogenated vegetable oil is associated with a variety of adverse outcomes, but little is known about the health effects of ruminant trans fats. Trans-vaccenic acid (TVA) is a naturally occurring TFA found in the fat of ruminants and in human dairy products. The present study was conducted to investigate the anticancer activity and underlying mechanisms of TVA on human nasopharyngeal carcinoma (NPC) 5-8F and CNE-2 cells.

**Methods:**

A CCK8 assay was used to determine the effect of TVA and the Mcl-1 inhibitor S63845 on the proliferation of NPC cells. Apoptosis was measured using flow cytometry. Western blotting was used to detect the protein expression levels of factors associated with Bcl-2-family protein signaling and Akt signaling.

**Results:**

TVA significantly inhibited cell proliferation in a dose-dependent manner. Mechanistic investigation demonstrated that TVA significantly decreased p-Akt levels and Bad phosphorylation on Ser-136 and Ser-112. More importantly, we discovered that the Mcl-1 inhibitor S63845 synergistically sensitized NPC cells to apoptosis induction by TVA.

**Conclusion:**

TVA can inhibit NPC cell growth and induced apoptosis through the inhibition of Bad/Akt phosphorylation. The combined use of TVA and Mcl-1 inhibitors offers a potential advantage for nasopharyngeal cancer treatment.

## Introduction

Trans fatty acids (TFAs) is a general term for unsaturated fatty acids with at least one double bond in the trans configuration [1]. TFAs within the human diet are mainly derived from industrial partial hydrogenation of vegetable oils and from natural sources, such as ruminant animal products. Evidence suggests that TFAs from different sources cause various biological effects on human health that may be beneficial or unfavorable [2, 3].

The impact of TFAs on the cardiovascular system has been extensively studied, and many epidemiological investigations and experiments have shown that TFAs from partially hydrogenated oils have adverse effects on the cardiovascular system [4]. TFAs formed via industrial hydrogenation could significantly accelerate the development of atherosclerosis by increasing the ratio of low-density lipoprotein (LDL) to high-density lipoprotein (HDL) [5]. In addition, many other studies have shown that these types of TFAs also have adverse effects on blood lipids [6], inflammation [7], oxidative stress [8], endothelial health [9], body weight [10], insulin sensitivity [11] and cancer [12]. However, emerging evidence indicates that trans fats derived from milk or ruminant body fats are beneficial for reducing the incidence of cardiovascular disease, cancer and obesity [13].

Trans-vaccenic acid (TVA) is ubiquitous in ruminant-derived fats and human dairy products such as milk and butter. It is worth noting that TVA is also the predominant TFA in human milk. Supplementation with milk lipids that contain TVA triggers a pronounced cytotoxic effect on HT29 cell due to conversion to c9,t11-conjugated linoleic acid (CLA) [14]. Suppression of tumor cell growth by TVA treatment of the MCF7 and SW480 cell lines can be attributed to the induction of apoptosis though increased DNA fragmentation and reduced cytosolic glutathione levels [15]. Preclinical studies have shown that the use of various types of fatty acids alone or combined with other anticancer drugs has promising therapeutic application prospects [16].

Nasopharyngeal carcinoma (NPC), the most common cancer originating in the nasopharynx, has a high incidence in Southern China and Southeast Asia [17]. Radiotherapy is currently the preferred method of treatment for early-stage NPC because most NPCs are poorly differentiated cancers with high sensitivity to radiation and because the primary and neck lymphatic drainage areas are easily included in the radiotherapy field [18]. Clinical treatment of recurrent or metastatic NPC is more difficult than primary NPC treatment. The routine treatment for these groups of patients is platinum-based chemotherapy, which confers a median progression-free survival time of 7 months [19]. Therefore, it is urgent to identify a more effective treatment option for patients with recurrent or metastatic NPC.

In the present study, we demonstrate that TVA effectively induces NPC apoptosis in 5-8F and CNE-2 cells. Mechanism studies indicate that TVA significantly inhibits Akt/Bad phosphorylation. More importantly, we found that TVA treatment also led to the upregulation of Mcl-1 as a novel mechanism involved in TVA resistance, which could be overcome by treatment with the Mcl-1 inhibitor S63845. These results suggest that the combination of TVA and Mcl-1 inhibitors is a promising approach for NPC treatment strategies.

## Materials and methods

### Cell culture and treatments

The human NPC cell lines 5-8F and CNE-2 were a generous gift from Prof. Chao-Nan Qian at the State Key Laboratory of Oncology in South China and the Collaborative Innovation Center for Cancer Medicine, Sun Yat-Sen University Cancer Center. All NPC cell lines were maintained in RPMI-1640 medium (SH30809.01, HyClone) supplemented with 10% FBS (10099–141, Gibco) and 1% penicillin-streptomycin (15070–063, Gibco) at 37 °C with 5% CO_2_.

For treatments, the cells were grown in 6-well plates to 60–70% confluence and were then treated with different concentrations of TFAs (EA, LA, and TVA; Nu-Chek Prep) (0, 25, 50, 100, 200 μM) for 24 h. Equal concentrations of DMSO were used as the controls.

### Cell viability assay

The effect of TFAs on the viability of NPC cells was measured by CCK8 assay. 5-8F and CNE-2 cells were plated (1.5 × 10^4^ cells) in 96-well culture plates and incubated in serum-containing medium for 24 h. The medium was then replaced with serum-free media containing different concentrations of TFA and/or S63845 (HY-100741, MedChem Express) for 24 h. After incubation, cell proliferation was analyzed with a CCK8 assay kit following the manufacturer’s instructions (CK04, Dojindo Laboratories), and the absorbance at 450 nm was measured using a microplate reader (BioTek).

### Annexin V and propidium iodide (PI) staining

The effects of the TFAs on apoptosis were determined by dual staining with annexin V-FITC and PI using an apoptosis detection kit from BD Bioscience. After treatment with the indicated TFA concentrations for 24 h, the cells were harvested and washed with PBS. The cells were then incubated with 5 μL of annexin V-FITC and PI for 15 min. The fluorescence of the cells was analyzed by flow cytometry (Beckman Coulter).

### Western blot assay

After treatment, lysates from the cultured cells were prepared with RIPA buffer, and the protein concentrations were determined with a BCA Protein Assay Reagent Kit (23227, Thermo Scientific). The lysates were separated by electrophoresis on a 12% SDS-polyacrylamide gel and then transferred to PVDF Western Blotting Membranes (IPVH00010, Millipore). The membranes were blocked with 5% milk and incubated at 4 °C with primary antibodies against Bad (9292, Cell Signaling Technology, 1:1000), phosphor-Bad (Ser-112) (5284, Cell Signaling Technology, 1:1000), phosphor-Bad (Ser-136) (4366, Cell Signaling Technology, 1:1000), Mcl-1 (94,296, Cell Signaling Technology, 1:1000), Bcl-xL (2764, Cell Signaling Technology, 1:1000), Bcl-2 (12789–1-AP, Proteintech, 1:1000), pan-Akt (4685, Cell Signaling Technology, 1:1000), p-Akt (Ser-473) (4060, Cell Signaling Technology, 1:1000), p-Akt (Thr-308) (13,038, Cell Signaling Technology, 1:1000), and β-tubulin (10068–1-AP, Proteintech, 1:5000). After overnight incubation with the primary antibodies, the membranes were incubated with secondary anti-mouse (115–035-003, Jackson, 1:5000) or anti-rabbit (111–035-003, Jackson, 1:5000) antibodies. Then, the signals were detected with an Amersham Imager 600.

### Calculation of the combination index

Whether the synergistic inhibitory effect between TVA and S63845 can be determined by the combination index (CI) using the Chou-Talalay equation [20]. 5-8F and CNE-2cells were treated with various concentrations of TVA and S63845 separately or in combination. The total inhibitory effect was assessed by CCK8 assay as described above. The CI value was determined as follow:

$$ CI=\frac{DA,x}{ICx,A}+\frac{DB,x}{ICx,B} $$

A, B represents two different agent, ICX, A and ICX, B are the concentration when the two agent are used alone to achieve a growth inhibition rate of X%, and DA,x and DB,x are the concentration of two agent combined to achieve a growth inhibition rate of X%. CI < 1 indicate synergism, CI = 1indicates additive effects, and CI > 1 indicates antagonism.

### Statistical analysis

Statistical analysis was performed using the standard Student t test for pair comparisons and ANOVA analysis for multiple factors. The statistical values of *P < 0.05, **P < 0.01 and ***P < 0.001 were considered statistically significant. Values of mean determinants are presented as ± s.e.m.

## Results

### TVA inhibits NPC cell viability in a dose-dependent manner

**Fig. 1:**
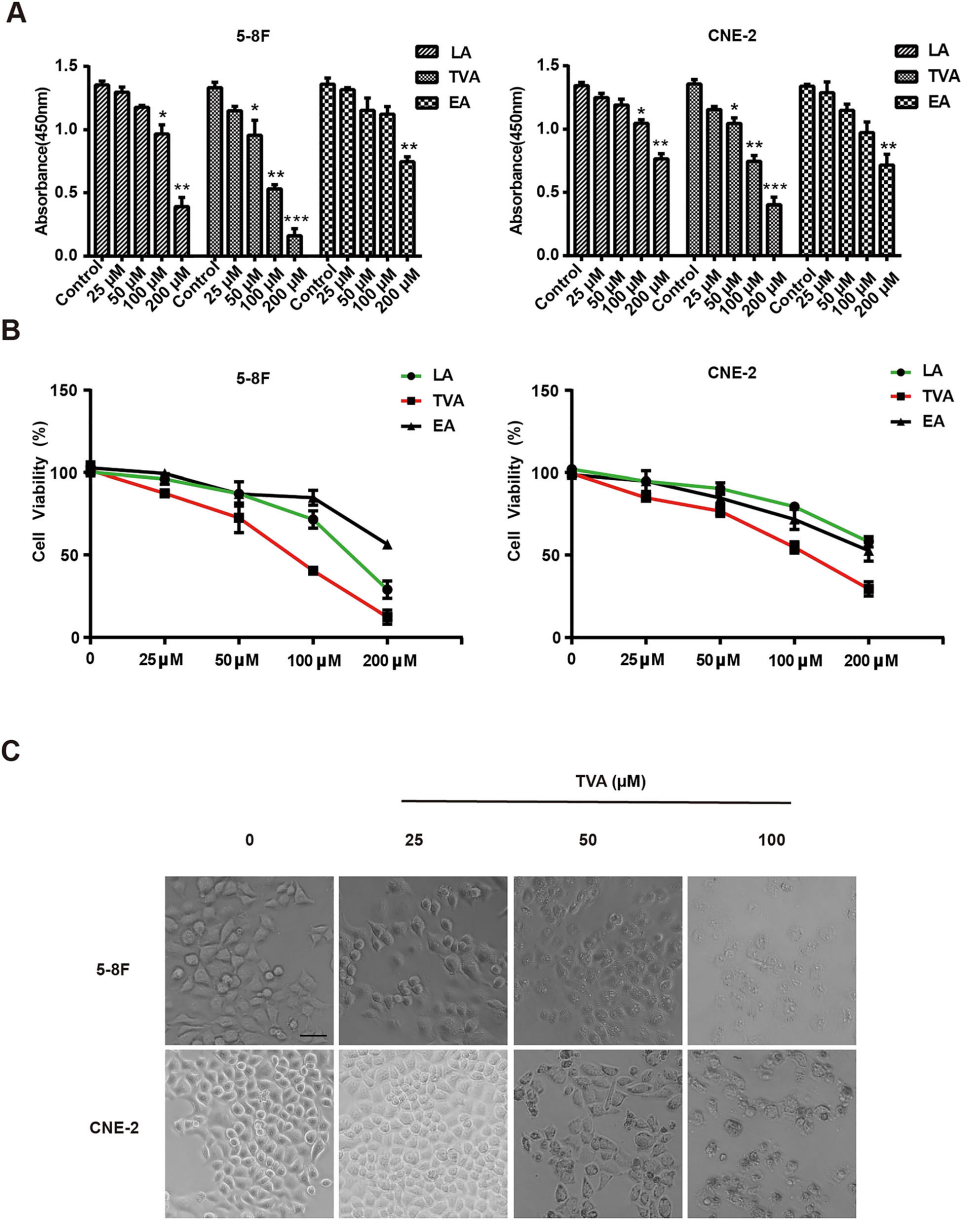
Effects of TVA on cell growth in human NPC cells. **a**. Optical density at 450 nm (OD450) values of NPC cells after TFA treatment for 24 h. **b**. Cell viability was assessed by CCK8 assays to calculate the survival rates. **c**. Cell morphology shrinkage was consistent with apoptotic cell death. *P<0.05, **P<0.01 and ***P<0.001 versus the control group

To determine the effect of TVA on NPC cells viability, we treated two human NPC cell line subtypes, 5-8F and CNE-2 (highly metastatic strain) with TFAs using a range of doses. After 24 h of treatment, cell viability was evaluated using a CCK8 assay. We found that all 3 TFAs exerted inhibitory effects on 5-8F and CNE-2 cell viability in a dose-dependent manner (Fig. 1a). The number of cells was significantly lower in the low-dose TVA (50 μM) group than in the control group, whereas 100 μM LA and 200 μM EA supplementation suppressed NPC cells viability to a significant degree. The growth inhibitory ratios of 5-8F cells treated with 25, 50, 100 and 200 μM TVA were 10.8, 18.9, 49.3 and 80.2%, respectively (*P* < 0.05), and those of CNE-2 cells were 7.9, 15.2, 45.3 and 70.5%, respectively (P < 0.05) (Fig. 1b). The cytotoxic effect of TVA was much greater in the 5-8F NPC cells (EC50 = 81.5 μM) than in CNE-2 cells (EC50 = 124 μM).

### TVA induces apoptosis in NPC cells in a dose-dependent manner

**Fig. 2:**
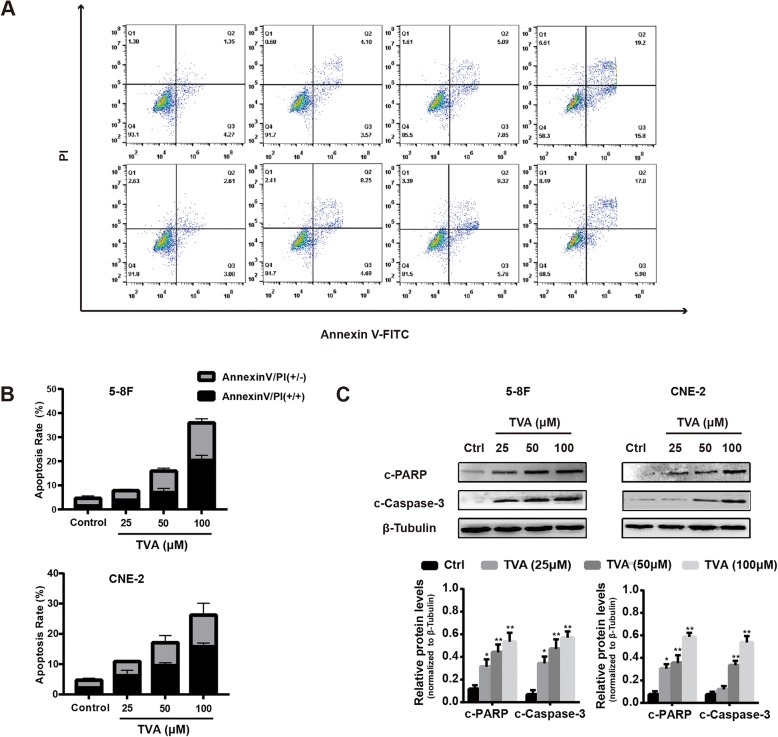
TVA treatment induces apoptosis in NPC cells. **a**. Apoptosis was analyzed by flow cytometry with PI and annexin V-FITC staining after 5-8F and CNE-2 cells were treated with TVA for 24 h at the indicated concentrations. **b**. The percentage of apoptotic cells was calculated as the apoptosis rate. **c**. Cleaved PARP (c-PARP) and cleaved caspase-3 (c-Caspase-3) protein levels after TVA treatment for 24 h. β-tubulin was used as an internal control. *P<0.05 and **P<0.01 versus the control group

To investigate whether apoptosis causes inhibition of cell growth, we analyzed apoptosis after TVA treatment by flow cytometry analysis of annexin V/PI-stained cells. We found that TVA significantly induced apoptosis in a dose-dependent manner. Incubation of 5-8F cells with 25, 50 or 100 μM TVA for 24 h caused 7.67, 12.9% or 35% increases in total apoptosis, respectively. Similarly, after incubation with 25 50 or 100 μM CNE-2, the percentage of apoptotic cells was increased by 12.9, 15.1% or 22.3%, respectively (Fig. 2a). Cleaved poly (ADP-ribose) polymerase (PARP) and cleaved caspase-3 are widely used to detect apoptosis in cells. Therefore, we assessed the protein levels of cleaved PARP and cleaved caspase-3 in the presence or absence of TVA treatment. Immunoblot analysis revealed that TVA treatment significantly increased the levels of cleaved PARP and cleaved caspase-3 in a time-dependent manner (Fig. 2c).

### TVA induces apoptosis in NPC cells through Akt and bad inactivation

**Fig. 3:**
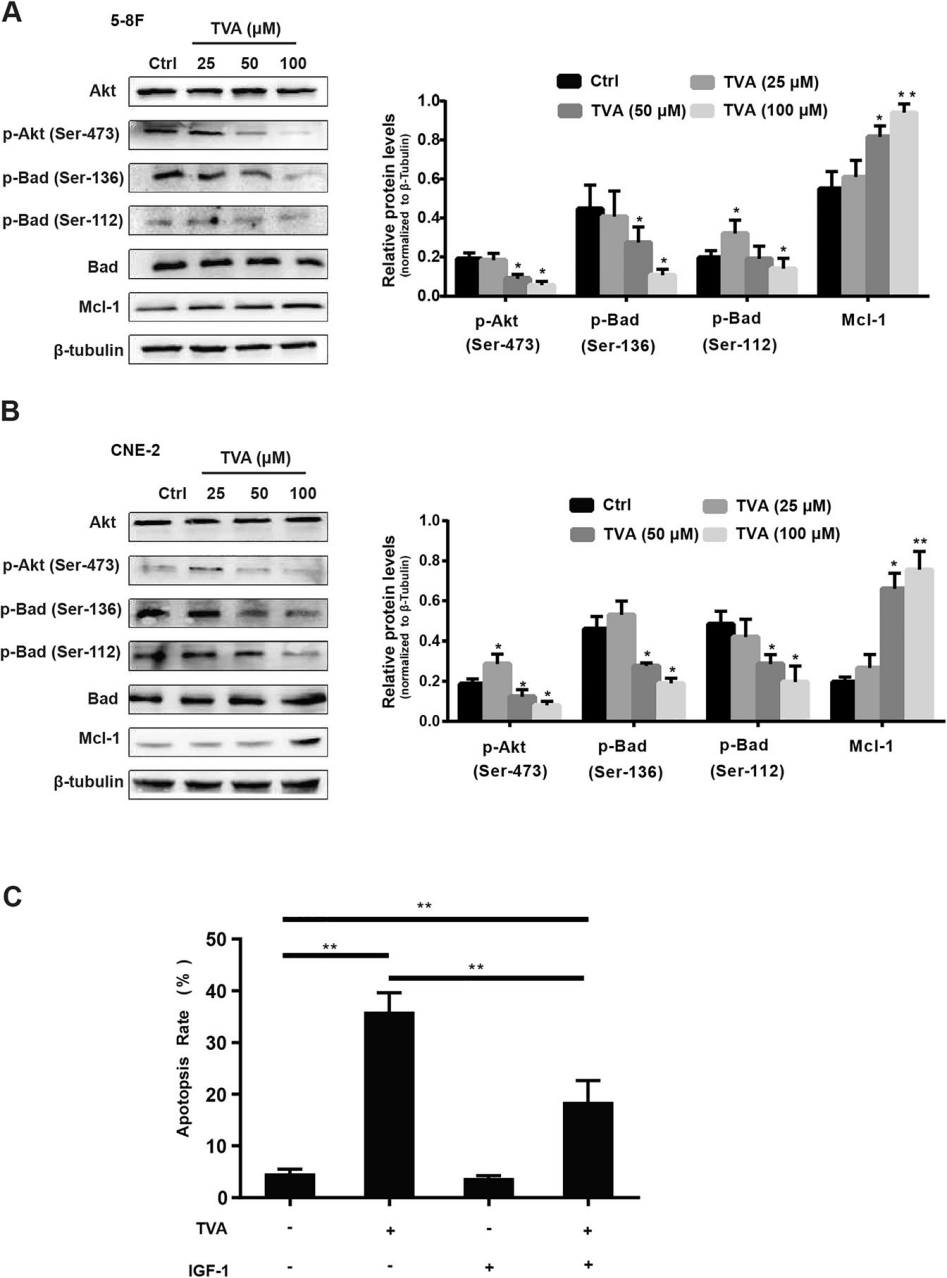
TVA induces apoptosis in NPC cells through Akt and Bad inactivation. **a**. The expression of Akt, p-Akt, Bad, p-Bad and Mcl-1 in 5-8F cells was analyzed by western blot assay after the cells were treated with TVA. **b**. The expression of Akt, p-Akt, Bad, p-Bad and Mcl-1 in CNE-2 cells was analyzed by western blot assay after the cells were treated with TVA. Protein expression was quantified by normalization to the level of β-tubulin. **c**. Effects of IGF-1 on TVA-induced apoptosis as detected by an annexin V-FITC/PI staining assay. The number of apoptotic cells was determined after treatment with TVA (100 μM) in the presence or absence of IGF-1 (50 ng/mL) for 24 h. The values represent the means±standard deviation of three independent experiments. *P<0.05 and **P<0.01 versus the control group

The Bcl-2-associated death promoter (Bad) is a pro-apoptotic member of the Bcl-2 family that can form a heterodimer with the antiapoptotic proteins Bcl-2 and Bcl-XL and prevent them from inhibiting apoptosis [21]. We were thus interested in determining whether TVA could affect endogenous Bad activity. The role of Bad in promoting apoptosis is mainly involves phosphorylation of Ser-136 and Ser-112. Bad is rapidly dephosphorylated and transferred to the mitochondria to induce apoptosis in response to external stimuli. As shown in Fig. 3a and b, TVA treatment induced a decrease in Bad phosphorylation on Ser-136 and Ser-112 in a concentration-dependent manner. Protein kinases such as Akt phosphorylate Bad at Ser136, thereby blocking Bad-induced apoptosis. We were further interested in determining whether the Akt pathway is involved in TVA-induced apoptosis. The expression levels of Akt in the TVA groups were not significantly different from those in the control group; however, the expression levels of p-Akt were significantly reduced in a dose-dependent manner compared to those in the control group, correlating closely with the findings regarding Bad Ser-136 phosphorylation. To confirm the role of Akt activation in apoptosis induction by TVA, we determined the effects of IGF-1, an activator of Akt. As shown in Fig. 3c, the combination of IGF-1 and TVA could neutralize the inhibitory effect of TVA alone. The above results indicate that the Akt/Bad pathway is involved in TVA-induced apoptosis.

### Synergistic inhibitory effect of combining TVA with S63845 in NPC cells

**Fig. 4:**
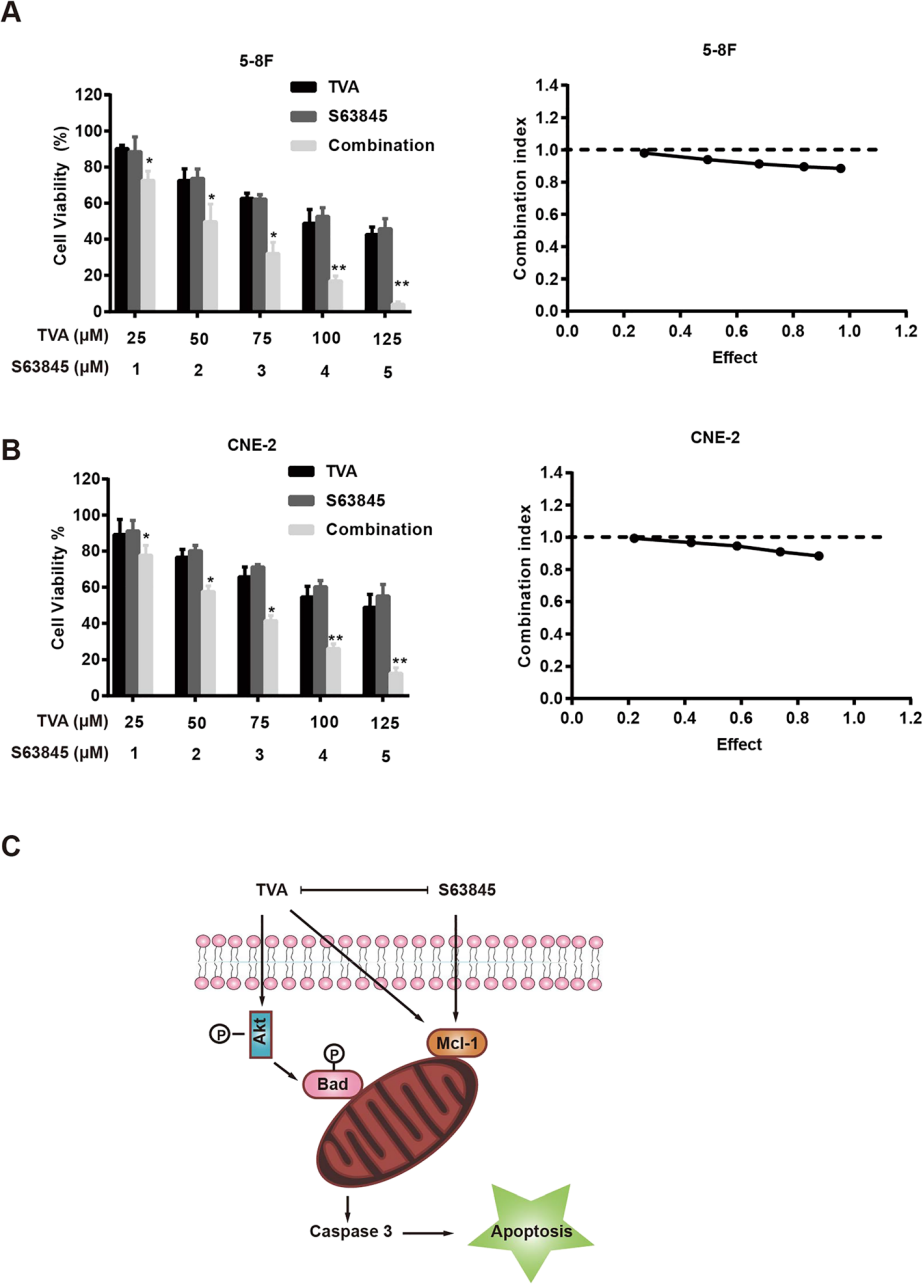
Synergistic inhibitory effect of the combination of TVA and S63845 on NPC cells. **a**. Cell viability of NPC cells after treatment with TVA (25, 50, 75, 100 and 125 μM), S683845 (1, 2, 3, 4 and 5 μM) or a combination of TVA and S63845 in a fixed ratio of 25:1 (TVA:S63845) for 24 h. **b**. CI value of the TVA/S63845 combination at each fixed ratio concentration. **c**. Signaling pathways underlying TVA and/or S63845-induced apoptosis in NPC cells

Mcl-1 is an anti-apoptotic member of the Bcl-2 family that inhibits apoptosis in response to a number of cytotoxic stimuli [22]. Mcl-1 is widely expressed in normal human tissues and is abnormally highly expressed in many malignant tumor tissues. As shown in Fig. 3a and b, TVA caused a significant increase in Mcl-1 expression in 5-8F and CNE-2 cells, which indicates that tumor cells are resistant to TVA. To verify our hypothesis, we treated cells with the Mcl-1 inhibitor S63845 combined with TVA and measured the overall inhibitory effects of the agents individually and in combination at a fixed ratio of 25:1 (TVA:S63845) using a CCK8 assay. As shown in Fig. 4a, S63845 treatment alone caused the inhibition of NPC cell growth, while the combination of TVA and S63845 was much more effectively than either alone. The CI value was less than 1 at all doses, suggesting that these two compounds have synergistic inhibitory effects on 5-8F and CNE-2 cells.

## Discussion

TFAs from different sources have unique biological effects. Many clinical and experimental studies have shown that the consumption of TFA from partially hydrogenated oils not only adversely affects the cardiovascular system but also accelerates the occurrence of diseases such as obesity and tumors [23, 24]. However, in recent years, increasing evidences has demonstrated that TFAs from ruminant trans fats have beneficial effects on human health [25]. In the present study, we examined the anticancer effects of TVA on human NPC 5-8F and CNE-2 cells and explored the related molecular mechanisms. Furthermore, we found that TVA treatment can promote a significant increase in Mcl-1 leading to drug resistance in NPC cells, which could be overcome by treatment with the Mcl-1 inhibitor S63845.

Apoptosis is an autonomous, ordered death of cells controlled by gene regulation. Biochemical events occur during this process, which is characterized by blebbing, cell shrinkage and nuclear fragmentation [26]. Apoptosis of cancer cells is an important mechanism to inhibit cell growth, and inducing tumor cell apoptosis has become the first choice for clinical anticancer therapy [27]. Therefore, we examined whether TVA could induce apoptosis in NPC cells in the present study. The CCK8, annexin V-FITC/PI staining, and western blot assay results revealed that the viability of 5-8F and CNE-2 cells was inhibited by TVA in the range of 25–100 μM in a dose-dependent manner (*P* < 0.05).

Bad is a pro-apoptotic member of the Bcl-2 family. Remarkably, dephosphorylated Bad, but not phosphorylated Bad, forms a heterodimer with the antiapoptotic proteins Bcl-2 and Bcl-XL, inactivating them and thus allowing apoptosis [28]. Phosphorylated Bad is located in the cytoplasm and has no pro-apoptotic activity [29]. Phosphorylation of Ser-112 and/or Ser-136 of Bad leads to the loss of proapoptotic activity [30]. Our results show that TVA can induce a concentration-dependent decrease in Bad phosphorylation at both Ser-136 and Ser-112. However, as a common protein in multiple signaling pathway, Bad is phosphorylated by several protein kinases such as those induced by survival signals. The Akt signaling pathway plays an important role in controlling tumor cell proliferation, the cell cycle and metastasis [31]. Furthermore, Akt, have been reported to phosphorylate Bad in response to survival signals [32]. IOur study, we found that the decrease in Bad phosphorylation was consistent with Akt-mediated dephosphorylation. Specific activation of Akt (by IGF-1) inhibited apoptosis and significantly attenuated the inhibitory effect of TVA. TVA promotes NPC apoptosis by targeting the Akt/Bad signaling pathway.

Tumor cells develop drug resistance for many reasons, among which apoptotic regulation of protein expression is an important factor [33]. In various types of tumor cells, anti-apoptotic genes of the Bcl-2 family are highly expressed, thereby preventing apoptosis [34]. Mcl-1 is a member of the antiapoptotic Bcl-2 family of proteins. Abundant evidence to suggests that Mcl-1 is an important cancer target [35]. The root cause of resistance to widely used anticancer drugs, including Bcl-2 inhibitors [36], paclitaxel [37], vincristine [37], and gemcitabine [38] is upregulation of the Mcl-1 level. In the present study, TVA treatment induced Mcl-1 expression. To inhibit drug resistance resulting from Mcl-1 upregulation, we used the newly developed Mcl-1 inhibitor S63845 combinated with TVA and showed that the two compounds have a synergistic effect. Therefore, targeting Mcl-1 is a rational strategy to improve the efficacy of TVA.

## Conclusions

The present investigation confirms that TVA exerts a significant anti-NPC effect on 5-8F and CNE-2 cells in vitro. The anticancer activity of TVA can be attributed to its inhibition of proliferation and its induction of apoptosis through the inhibition of Bad/Akt phosphorylation. However, the increased expression of Mcl-1 will partly compromise the efficacy of TVA. Thus, the combined use of TVA and Mcl-1 inhibitors is a promising prospect for nasopharyngeal cancer treatment.

## Abbreviations

CI: Combination index
EA: Elaidic acid
HDL: High-density lipoprotein
LA: Linoelaidic acid
LDL: Low-density lipoprotein
NPC: Nasopharyngeal carcinoma
TFAs: Trans fatty acids
TVA: Trans-vaccenic acid
